# Adjunctive brexpiprazole in patients with unresolved symptoms of depression on antidepressant treatment who are early in the disease course: post hoc analysis of randomized controlled trials

**DOI:** 10.1093/ijnp/pyaf050

**Published:** 2025-07-03

**Authors:** Shivani Kapadia, Zhen Zhang, Csilla Csoboth, Mehul Patel, Michael E Thase, George I Papakostas

**Affiliations:** Otsuka Pharmaceutical Development & Commercialization Inc., Princeton, NJ, United States; Otsuka Pharmaceutical Development & Commercialization Inc., Princeton, NJ, United States; Medical Affairs, Lundbeck LLC, Deerfield, IL, United States; Otsuka Pharmaceutical Development & Commercialization Inc., Princeton, NJ, United States; Perelman School of Medicine, University of Pennsylvania and the Philadelphia Veterans Affairs Medical Center, Philadelphia, PA, United States; Department of Psychiatry, Clinical Trials Network and Institute, Massachusetts General Hospital, Harvard Medical School, Boston, MA, United States

**Keywords:** brexpiprazole, depression, antidepressant, disease course, treatment optimization

## Abstract

**Background:**

Treatment for major depressive disorder (MDD) should be optimized as early as possible in the disease course to minimize patient suffering and maximize clinical benefits. This post hoc analysis aimed to investigate the efficacy and safety of adjunctive brexpiprazole in patients who were earlier and later in the disease course.

**Methods:**

Data were pooled from three 6-week, randomized, double-blind, placebo-controlled trials of adjunctive brexpiprazole in adult outpatients with MDD and inadequate response to antidepressant treatment. “Earlier” and “later” disease course subgroups were defined based on the proxies of median age, age at diagnosis, number of episodes, episode duration, and number of prior antidepressants. Efficacy was assessed by changes in Montgomery–Åsberg Depression Rating Scale (MADRS) total score, and safety by treatment-emergent adverse events.

**Results:**

Greater improvement in MADRS total score at week 6 (*P* < .05) was observed for antidepressant + brexpiprazole 2–3 mg/day (*n* = 579) versus antidepressant + placebo (*n* = 583) in all subgroups representing earlier and later disease course, with treatment effects (least-squares mean differences in score change) ranging from −1.79 to −2.92 points. The incidence of treatment-emergent adverse events across subgroups was 53.1%–67.2% for antidepressant + brexpiprazole 2–3 mg/day and 43.0%–51.8% for antidepressant + placebo, with no consistent differences in patients who were earlier or later in the disease course.

**Conclusions:**

Adjunctive brexpiprazole improved depressive symptoms earlier in the disease course, when benefits to patients and healthcare systems can be maximized. Adjunctive brexpiprazole also improved depressive symptoms later in the disease course; there was no advantage of delaying brexpiprazole treatment.

**Trial registration:**

Post hoc analysis of NCT01360645, NCT01360632, NCT02196506 (ClinicalTrials.gov).

Significance Statement Only around half of patients with depression respond to initial antidepressant treatment, and it can take multiple different treatment steps to find the right treatment for a particular patient. It is important to find the right treatment as early as possible to provide patients with symptom relief. One treatment option for patients with unresolved symptoms is to take brexpiprazole as well as an antidepressant. The aim of our study was to investigate whether adding brexpiprazole to antidepressant treatment improves symptoms in groups of patients who have been living with depression for a shorter time, and for a longer time. We found that adding brexpiprazole helped patients regardless of how long they had lived with depression. In general, the earlier that patients receive the right treatment, the longer they will benefit from it.

## INTRODUCTION

Depressive disorders affect 332 million people worldwide, are a leading cause of disability, increase the risk of all-cause mortality, and exert a substantial economic burden.^1-4^ Guidelines recommend various psychological and pharmacological therapies for depression, of which antidepressant treatment is first-line pharmacotherapy (commonly a selective serotonin reuptake inhibitor [SSRI] or serotonin–noradrenaline reuptake inhibitor [SNRI]).^5-7^ However, only around 50% of patients with major depressive disorder (MDD) respond to initial antidepressant treatment,^8^ and unresolved symptoms are common.^9^ Thus, for some patients, multiple steps may be required to optimize MDD treatment.^10^

Options for patients with unresolved symptoms of depression include switching antidepressants, combining antidepressants, or augmenting with another drug such as an atypical antipsychotic (among other options).^5-7^ However, repeated “trial-and-error” of different treatments can result in patients feeling frustrated and dissatisfied, increasing the risk of adherence issues.^11^ Optimized treatment should therefore be provided as early in the disease course as possible to increase the likelihood of achieving full symptomatic and functional recovery.^10^

The 3 main monoamine systems associated with the pathophysiology of depression are noradrenaline (norepinephrine), serotonin, and dopamine.^12^^,^^13^ Brexpiprazole is an atypical antipsychotic with subnanomolar affinity for receptors across these 3 monoaminergic systems.^14^ Four phase 3 randomized controlled trials have demonstrated the efficacy and tolerability of adjunctive brexpiprazole in patients with MDD and unresolved symptoms on antidepressant treatment.^15-18^ Given that outcomes can be improved by shortening the time from diagnosis to optimized treatment,^10^ it is hypothesized that patients with unresolved symptoms on antidepressants may benefit from adjunctive brexpiprazole early in the disease course. The aim of this post hoc analysis was to investigate the efficacy and safety of adjunctive brexpiprazole in patients who were earlier and later in the disease course (based on the proxies of age, age at diagnosis, number of lifetime episodes, episode duration, and number of prior antidepressants), using pooled data from randomized controlled trials.

## METHODS

### Study Design and Patients

Data were included from 3 phase 3 randomized controlled trials (ClinicalTrials.gov identifiers: NCT01360645, NCT01360632, NCT02196506) conducted at multiple sites in North America, Europe, and Russia between June 2011 and May 2016.^15-17^ The fourth phase 3 trial was not included in this pooled analysis because it had a different design from the other 3 trials.^18^ Each of the trials was conducted in accordance with the Declaration of Helsinki, the International Conference on Harmonisation Good Clinical Practice guidelines, and local regulatory requirements. The trials were approved by relevant institutional review boards or independent ethics committees, and all patients provided written informed consent prior to enrollment.

The 3 trials had similar eligibility criteria and designs, which have previously been published.^15-17^ In brief, the trials enrolled adult outpatients (18–65 years) with a Diagnostic and Statistical Manual of Mental Disorders, Fourth Edition, Text Revision (DSM-IV-TR) diagnosis of single or recurrent, nonpsychotic MDD and a current depressive episode of ≥ 8 weeks in duration. Patients were required to have a history of inadequate response (<50% improvement based on self-report) to an adequate trial of 1–3 prior antidepressants during the current episode. Patients were ineligible if they presented with suicidal ideation or behavior, substance abuse or dependence, or other protocol-specified psychiatric comorbidities.

The trials had 2 distinct treatment periods. Initially, patients received an investigator-determined, open-label SSRI/SNRI antidepressant (escitalopram, fluoxetine, paroxetine controlled release, sertraline, duloxetine, or venlafaxine extended release) + single-blind placebo for 8 weeks. The purpose of this period was to identify patients with inadequate response to antidepressant treatment (ie, persistent symptoms without substantial improvement) under clinical trial conditions. Those patients who met protocol-specified criteria for inadequate response based on clinician-reported depression rating scale scores entered a randomized, double-blind, placebo-controlled, parallel-arm treatment period. In this period, patients received the same antidepressant + either adjunctive brexpiprazole or adjunctive placebo for 6 weeks. Depending on the trials, brexpiprazole was dosed at 1, 2, or 3 mg/day (fixed doses) with titration over 1–2 weeks.

### Definition of Earlier and Later Disease Course

In this post hoc analysis, patients were stratified into “earlier” versus “later” disease course subgroups according to baseline demographic and clinical characteristics. The following 5 characteristics were considered relevant, for a total of 10 subgroups.

1. Age. Clinical trial data indicate that duration of illness (ie, time since diagnosis of MDD) increases with age,^19^ suggesting that older patients may be later in the disease course than younger patients. In the present analysis, the earlier disease subgroup was defined as patients with age < median, and the later disease subgroup as patients with age ≥ median.
2. Age at diagnosis of MDD. In the US-nationwide STAR^*^D (Sequenced Treatment Alternatives to Relieve Depression) study, younger age at onset was associated with a longer duration of illness, before and after adjustment for current age,^20^ suggesting that patients with younger age at onset may be later in the disease course than patients with older age at onset. In the present analysis, the earlier disease subgroup was defined as patients with age at diagnosis ≥ median, and the later disease subgroup as patients with age at diagnosis < median.
3. Number of lifetime depressive episodes. In the STAR^*^D study, a higher number of depressive episodes was associated with a longer duration of illness, before and after adjustment for age.^21^ In the present analysis, the earlier disease subgroup was defined as patients with < median number of depressive episodes, and the later disease subgroup as patients with ≥ median number of depressive episodes.
4. Duration of current depressive episode. Longer episode duration has been linked with younger age at onset of depression,^22^ and with decreased likelihood of remission and recovery.^23^^,^^24^ In the present analysis, the earlier disease subgroup was defined as patients with < median duration of current depressive episode, and the later disease subgroup as patients with ≥ median duration of current depressive episode.
5. Number of prior antidepressants in the current episode. In the STAR^*^D study, patients with a current depressive episode were administered sequential treatments until they experienced an acceptable benefit, preferably achieving symptom remission.^25^ A greater number of treatment steps was associated with a longer duration of illness at baseline,^25^ suggesting that patients with a greater number of prior treatments in the current episode may be later in the disease course than patients with fewer prior treatments. In the brexpiprazole trials, patients with 1–3 prior antidepressants at screening were eligible to enroll, and approximately 80% of randomized patients had inadequate response to 1 prior antidepressant at screening.^26^ Thus, in the present analysis, the earlier disease subgroup was defined as patients with 1 prior antidepressant at screening, and the later disease subgroup as patients with 2–3 prior antidepressants at screening (As described in the “Study Design” section, to be eligible for randomization, patients were required to have inadequate response to an additional open-label antidepressant after screening).

### Outcome Measures

The primary efficacy measure in each trial was the Montgomery–Åsberg Depression Rating Scale (MADRS), a clinician-rated measure of depressive symptom severity that is widely used in MDD trials.^27^ The MADRS comprises 10 items (apparent sadness, reported sadness, inner tension, reduced sleep, reduced appetite, concentration difficulties, lassitude, inability to feel, pessimistic thoughts, and suicidal thoughts), each of which is rated on a 7-point scale from 0 (least severe) to 6 (most severe). Thus, total scores range from 0 (least severe) to 60 (most severe).

The Clinical Global Impressions—Severity of illness (CGI-S) scale was included as a secondary efficacy measure.^28^ The CGI-S is a clinician-rated, single-item measure of overall illness severity, rated from 1 (normal, not at all ill) to 7 (among the most extremely ill patients).

In the brexpiprazole trials, safety was assessed by a variety of standard subjective and objective measures, including the incidence of patient-reported treatment-emergent adverse events (TEAEs).^15-17^ In addition to the overall incidence of TEAEs, the present analysis investigated the incidence of extrapyramidal-symptom-related TEAEs (including akathisia, dyskinesia, dystonia, and parkinsonism) and increased body weight as a TEAE, which are of particular interest in relation to antipsychotic treatment.

### Data Analysis

The focus of this analysis was the 2–3 mg/day dose of brexpiprazole, which comprises the recommended dose (2 mg/day) and the maximum dose (3 mg/day, permitted in the United States) for the adjunctive treatment of MDD.^29^^,^^30^ Data for patients randomized to brexpiprazole 2 or 3 mg were pooled from all 3 trials. A corresponding pooled 2 or 3 mg-equivalent placebo group was created. As a supporting analysis, a pooled brexpiprazole 2 mg/day group was also investigated, representing the recommended dose for the adjunctive treatment of MDD,^29^^,^^30^ with a corresponding 2 mg-equivalent placebo group. The 2 mg analysis included data from only the 2 trials that investigated the 2 mg dose, and therefore comprised fewer patients than the 2–3 mg analysis. All analyses were performed in the sample of patients randomized as per final protocols who received at least one dose of double-blind medication and who had a baseline and at least one post-baseline MADRS total score evaluation recorded in the randomized treatment period. Baseline values were defined as the last values obtained prior to randomization.

Least-squares mean changes from baseline in MADRS total score and CGI-S score were calculated using a mixed model for repeated measures (MMRM) in an observed-cases dataset with no imputation for missing data. Treatment effect was calculated as the least-squares mean difference in score change between antidepressant + brexpiprazole and antidepressant + placebo at week 6. The MMRM had factors of treatment, visit, site nested in trial, baseline, and interaction of treatment with visit, as well as interaction of baseline with visit. An unstructured covariance was used by default. Between-group comparisons were tested at a nominal 0.05 level (2-sided), with no adjustment for multiplicity.

Patient baseline demographic and clinical characteristics, and the incidence of TEAEs, were summarized using descriptive statistics.

Analyses were performed using SAS Enterprise Guide 8.2 (SAS Institute Inc, Cary, NC, USA).

## RESULTS

### Patients

In the 2–3 mg analysis, data were analyzed for 579 patients who received antidepressant + brexpiprazole 2–3 mg/day, and 583 who received antidepressant + placebo. Completion rates were 539/579 (93.1%) for antidepressant + brexpiprazole 2–3 mg/day and 557/583 (95.7%) for antidepressant + placebo. The most common reasons for discontinuation (>1% for antidepressant + brexpiprazole 2–3 mg/day or antidepressant + placebo, respectively) were patient withdrawal of consent (13 [2.2%], 13 [2.2%]) and adverse events (17 [2.9%], 3 [0.5%]). In the 2 mg analysis, data were analyzed for 366 patients who received antidepressant + brexpiprazole 2 mg/day, and 380 who received antidepressant + placebo. Completion rates were 341/366 (93.2%) for antidepressant + brexpiprazole 2 mg/day and 363/380 (95.5%) for antidepressant + placebo. The most common reasons for discontinuation (>1% for antidepressant + brexpiprazole 2 mg/day or antidepressant + placebo, respectively) were patient withdrawal of consent (10 [2.7%], 7 [1.8%]) and adverse events (9 [2.5%], 1 [0.3%]).

Baseline demographic and clinical characteristics were similar between treatment groups (see Table 1 for the 2–3 mg analysis, and Table S1 for the 2 mg analysis).

**Table 1:** Baseline demographic and clinical characteristics and assigned antidepressant treatments (2–3 mg analysis).

**Characteristic**	**ADT + placebo (*n* = 583)**	**ADT + brexpiprazole 2–3 mg (*n* = 579)**
**Age (years), mean (SD)**	44.7 (11.9)	43.9 (11.8)
**Sex, *n* (%)**		
**Female**	402 (69.0)	412 (71.2)
**Male**	181 (31.0)	167 (28.8)
**BMI (kg/m^2^), mean (SD)**	29.6 (7.1)	29.7 (6.8)
**Race, *n* (%)**		
**White**	499 (85.6)	502 (86.7)
**Other**^a^	84 (14.4)	77 (13.3)
**Duration of current episode (months), mean (SD)**	17.1 (36.2)	15.1 (23.7)
**Number of lifetime episodes, mean (SD)**	3.5 (3.2)	3.4 (2.6)
**Number of prior ADTs at screening, *n* (%)** ^b^		
**1**	469 (80.4)	467 (81.5)
**2**	101 (17.3)	93 (16.2)
**3**	13 (2.2)	13 (2.3)
**MADRS total score, mean (SD)**	26.6 (5.7)	26.8 (5.5)
**CGI-S score, mean (SD)**	4.2 (0.6)	4.2 (0.6)
**Assigned ADT, *n* (%)**		
**Escitalopram**	113 (19.4)	113 (19.5)
**Fluoxetine**	91 (15.6)	86 (14.9)
**Paroxetine CR**	61 (10.5)	72 (12.4)
**Sertraline**	90 (15.4)	78 (13.5)
**Duloxetine**	122 (20.9)	129 (22.3)
**Venlafaxine XR**	106 (18.2)	101 (17.4)

a Including American Indian or Alaska Native, Asian, Black or African American, Native Hawaiian or Other Pacific Islander, and other non-specified (US Census Bureau classifications).

b Number of prior ADTs at screening was missing for 6 patients.

Abbreviations: ADT, antidepressant treatment; BMI, body mass index; CGI-S, Clinical Global Impressions—Severity of illness; CR, controlled release; MADRS, Montgomery–Åsberg Depression Rating Scale; SD, standard deviation; XR, extended-release.

Characteristics used to stratify patients into earlier and later disease course subgroups were as follows:

- Median age at baseline: 46 years (2–3 mg analysis) or 44.5 years (2 mg analysis).
- Median age at MDD diagnosis: 44 years (2–3 mg analysis) or 43 years (2 mg analysis).
- Median number of lifetime depressive episodes: 3 (both analyses).
- Median duration of current episode: 8 months (both analyses).
- Number of prior antidepressants: the majority of patients (>80%) had 1 prior antidepressant at screening (both analyses), supporting the chosen subgroups (1 or 2–3).

At baseline, MADRS total score was similar across treatment groups and disease course subgroups, except that the score was slightly higher among patients with longer duration of current episode (see Table 2 for the 2–3 mg analysis and Table S2 for the 2 mg analysis).

**Table 2:** MADRS total score at baseline and treatment effect at week 6, stratified by variables indicative of earlier and later disease course (2–3 mg analysis).

	**N**		**Mean (SD) at baseline**		**Treatment difference at week 6** **(ADT + brex vs ADT + placebo)**	
**Subgroup**	**ADT + placebo**	**ADT + brex 2–3 mg**	**ADT + placebo**	**ADT + brex 2–3 mg**	**LS mean (95% CI)**	***P*-value**
**Age at baseline**						
** < 46 years (earlier disease)**	283	297	26.5 (5.7)	26.5 (5.4)	−2.17 (−3.52 to −0.82)	.002
** ≥ 46 years (later disease)**	300	282	26.8 (5.7)	27.1 (5.6)	−2.40 (−3.82 to −0.97)	.001
**Age at diagnosis**						
** ≥ 44 years (earlier disease)**	305	285	26.7 (5.7)	26.9 (5.6)	−2.36 (−3.43 to −1.29)	<.001
** < 44 years (later disease)**	278	294	26.6 (5.8)	26.7 (5.5)	−2.30 (−3.66 to −0.95)	.001
**Number of lifetime episodes**						
** < 3 episodes (earlier disease)**	236	235	26.6 (5.9)	26.7 (5.4)	−1.79 (−3.31 to −0.26)	.022
** ≥ 3 episodes (later disease)**	347	344	26.6 (5.6)	26.9 (5.6)	−2.59 (−3.61 to −1.57)	<.001
**Duration of current episode**						
** < 8 months (earlier disease)**	272	256	25.8 (5.7)	25.6 (5.6)	−1.95 (−3.39 to −0.51)	.008
** ≥ 8 months (later disease)**	311	323	27.4 (5.6)	27.7 (5.3)	−2.92 (−4.05 to −1.79)	<.001
**Number of prior ADTs**						
** 1 prior ADT (earlier disease)**	469	467	26.5 (5.6)	26.9 (5.6)	−2.26 (−3.32 to −1.20)	<.001
** 2–3 prior ADTs (later disease)**	114	106	27.3 (6.2)	26.3 (5.3)	−2.77 (−4.83 to −0.70)	.009

Abbreviations: ADT, antidepressant treatment; brex, brexpiprazole; CI, confidence interval; LS, least squares; MADRS, Montgomery–Åsberg Depression Rating Scale; SD, standard deviation.

### Efficacy

**Figure 1:**
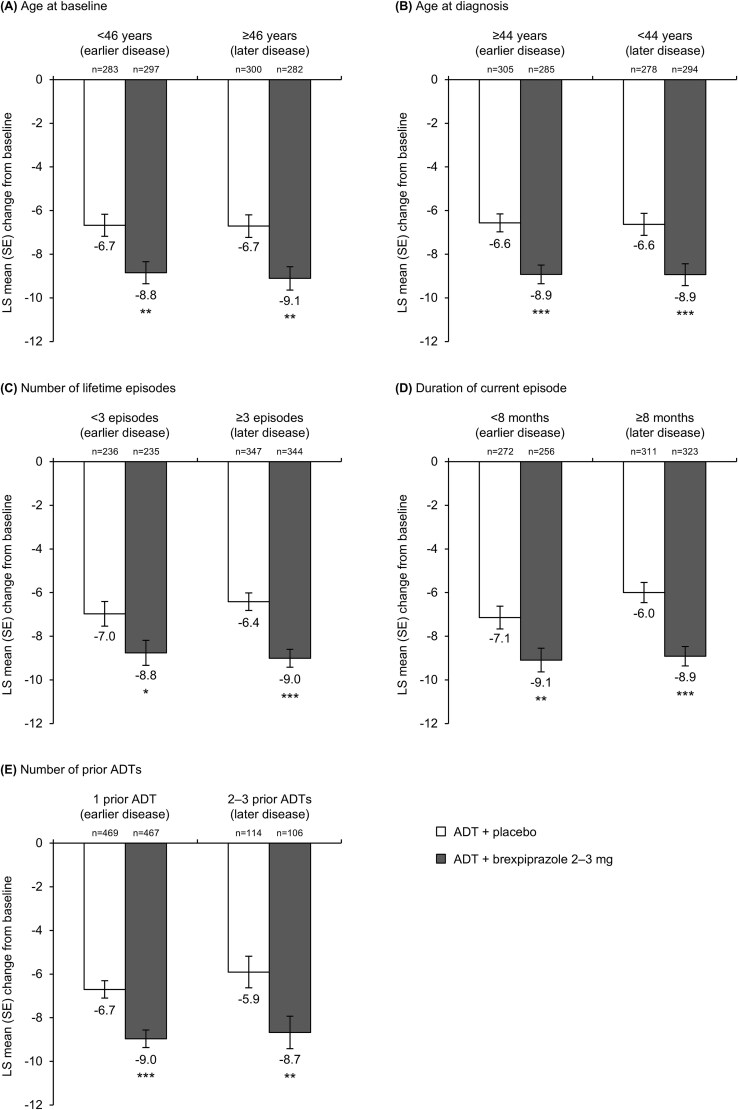
Mean change from baseline to week 6 in MADRS total score, stratified by variables indicative of earlier and later disease course (2–3 mg analysis). ^*^P < .05, ^**^P < .01, ^***^P < .001 versus ADT + placebo. *N*-values are for baseline. Abbreviations: ADT, antidepressant treatment; LS, least squares; MADRS, Montgomery–Åsberg Depression Rating Scale; SE, standard error.

For the 2–3 mg analysis, change from baseline to week 6 in MADRS total score is presented in Figure 1 according to age at baseline, age at diagnosis, number of lifetime episodes, duration of current episode, and number of prior antidepressants at screening. Greater improvement (*P* < .05) was observed with antidepressant + brexpiprazole 2–3 mg versus antidepressant + placebo in all subgroups, whether representing earlier disease course or later disease course. Treatment effects (least-squares mean differences in score change) at week 6 were −1.79 to −2.92 points across subgroups (Table 2). Changes from baseline by week are presented in Figure S1.

The CGI-S score changes from baseline to week 6 showed greater improvement (*P* < .05) with antidepressant + brexpiprazole 2–3 mg versus antidepressant + placebo in most subgroups representing earlier disease course and later disease course, with treatment effects at week 6 of −0.17 to −0.30 points (data not shown). The exception was the subgroup with < 3 lifetime depressive episodes (representing earlier disease course), which showed a numerical improvement with antidepressant + brexpiprazole (treatment effect, −0.13; *P* = .19).

Similar results were observed in the 2 mg analyses. The MADRS total score change is presented in Figure S2 and Table S2.

### Safety

Across subgroups, the proportion of patients with at least one TEAE ranged from 53.1%–67.2% for antidepressant + brexpiprazole 2–3 mg/day, and 43.0%–51.8% for antidepressant + placebo (Table 3). The incidence of TEAEs was not consistently higher or lower in patients who were earlier or later in the disease course. Similarly, the proportions of patients with at least one extrapyramidal-symptom-related TEAE and with increased body weight as a TEAE were greater for antidepressant + brexpiprazole 2–3 mg/day than antidepressant + placebo, and the incidence was not consistently higher or lower in patients who were earlier or later in the disease course (Table 3).

**Table 3:** Summary of TEAEs, stratified by variables indicative of earlier and later disease course (2–3 mg analysis).

	***N***		**≥1 TEAE**		**≥1 EPS-related TEAE**		**Increased body weight TEAE**	
**Subgroup**	**ADT + placebo**	**ADT + brex 2–3 mg**	**ADT + placebo**	**ADT + brex 2–3 mg**	**ADT + placebo**	**ADT + brex 2–3 mg**	**ADT + placebo**	**ADT + brex 2–3 mg**
**Age at baseline**								
** < 46 years (earlier disease)**	283	297	140 (49.5)	190 (64.0)	16 (5.7)	52 (17.5)	6 (2.1)	17 (5.7)
** ≥ 46 years (later disease)**	300	282	135 (45.0)	163 (57.8)	14 (4.7)	33 (11.7)	3 (1.0)	19 (6.7)
**Age at diagnosis**								
** ≥ 44 years (earlier disease)**	305	285	139 (45.6)	159 (55.8)	14 (4.6)	34 (11.9)	5 (1.6)	18 (6.3)
** < 44 years (later disease)**	278	294	136 (48.9)	194 (66.0)	16 (5.8)	51 (17.3)	4 (1.4)	18 (6.1)
**Number of lifetime episodes**								
** < 3 episodes (earlier disease)**	236	235	116 (49.2)	137 (58.3)	16 (6.8)	39 (16.6)	4 (1.7)	13 (5.5)
** ≥ 3 episodes (later disease)**	347	344	159 (45.8)	216 (62.8)	14 (4.0)	46 (13.4)	5 (1.4)	23 (6.7)
**Duration of current episode**								
** < 8 months (earlier disease)**	272	256	117 (43.0)	136 (53.1)	21 (7.7)	32 (12.5)	5 (1.8)	16 (6.3)
** ≥ 8 months (later disease)**	311	323	158 (50.8)	217 (67.2)	9 (2.9)	53 (16.4)	4 (1.3)	20 (6.2)
**Number of prior ADTs**								
** 1 prior ADT (earlier disease)**	469	467	216 (46.1)	280 (60.0)	21 (4.5)	63 (13.5)	8 (1.7)	26 (5.6)
** 2–3 prior ADTs (later disease)**	114	106	59 (51.8)	68 (64.2)	9 (7.9)	21 (19.8)	1 (0.9)	8 (7.5)

Data are *n* (%).

Abbreviations: ADT, antidepressant treatment; brex, brexpiprazole; EPS, extrapyramidal symptom; TEAE, treatment-emergent adverse event.

Similar results were observed in the 2 mg analyses (Table S3).

## DISCUSSION

In MDD, a patient’s treatment should be optimized as early as possible to provide symptom relief, maximize their opportunity for full functional recovery, and help prevent the cumulative damage and neuroplastic changes that may occur with repeated episodes of depression.^10^^,^^31-33^ In this post hoc analysis, adjunctive brexpiprazole 2–3 mg/day was associated with greater improvements in depressive symptoms than adjunctive placebo in patients with unresolved symptoms on antidepressants who were early in the disease course (based on the proxies of age, age at diagnosis, number of lifetime episodes, duration of current episode, and number of prior antidepressants). Similar improvements were observed in the brexpiprazole 2 mg/day analysis. These results suggest that adjunctive brexpiprazole may be a valuable treatment option when administered early in the disease course. Furthermore, this analysis showed that there was no advantage for patients in delaying adjunctive brexpiprazole treatment, since adjunctive brexpiprazole was efficacious to a similar degree in patients who were later in the disease course. Thus, patients with unresolved symptoms on antidepressants may obtain the maximum benefit from adjunctive brexpiprazole when it is initiated early in the disease course.^10^^,^^33^ Real-world evidence supports the early initiation of adjunctive antipsychotic treatment in patients with MDD to reduce healthcare resource use and costs.^34^^,^^35^ Specifically, initiation of adjunctive brexpiprazole within the first 2 months of antidepressant treatment (versus after 1 year) is estimated to reduce the annual cost of MDD-specific outpatient/inpatient visits and pharmacy by approximately 4000 USD per patient.^36^

Safety was assessed by the incidence of TEAEs, extrapyramidal symptoms (including akathisia, dyskinesia, dystonia, and parkinsonism), and weight increase. Given that MDD is a risk factor for the development and worsening of a range of comorbidities,^37^ it might be expected that patients later in the disease course will experience more medication side effects; however, this was only consistently observed for patients with a longer duration of current episode. With regard to treatment, adjunctive brexpiprazole was associated with a greater incidence of extrapyramidal symptoms and weight increase than adjunctive placebo, as previously noted in a pooled analysis of phase 3 trials.^26^ Prior studies indicate that adjunctive brexpiprazole is neither among the most activating (akathisia, restlessness, anxiety, and insomnia) nor the most sedating atypical antipsychotics^38^ and is associated with small changes in metabolic parameters and moderate weight gain during short- and long-term treatment.^39^^,^^40^

Strengths of this analysis are the large dataset that included over a thousand patients, and the use of 5 different approaches to identify patients early in the disease course. Limitations are the post hoc nature of the analysis with no correction for multiple comparisons, meaning that the results should be considered hypothesis-generating. With regard to the proxies for disease course, investigators may not accurately record number of lifetime episodes or duration of current episode when enrolling patients. There was no adjunctive active comparator in this analysis, and to the authors’ knowledge no other adjunctive antipsychotics have been investigated in these disease course subgroups, which means that comparisons with other agents cannot be made. Finally, as with all analyses of clinical trial data, patient selection criteria and other protocol-specified restrictions limit the generalizability of these results to a broader patient population.

## CONCLUSION

In patients with MDD and unresolved symptoms of depression on antidepressant treatment, therapeutic doses of adjunctive brexpiprazole improved depressive symptoms in patients who were earlier in the disease course, as well as those who were later in the disease course. Adjunctive brexpiprazole is generally well tolerated in MDD, and there were no consistent differences in adverse events in patients who were earlier or later in the disease course. These results suggest that eligible patients will benefit from adjunctive brexpiprazole regardless of their stage of MDD. However, treatment should be optimized as early as possible in the disease course to minimize patient suffering and maximize clinical benefits, as well as to reduce healthcare resource use and costs. Given that there was no advantage of delaying brexpiprazole treatment, adjunctive brexpiprazole may be of maximum benefit to patients and healthcare systems when taken early in the disease course.

## Supplementary Material

Supplement_22-May-25_pyaf050

## Data Availability

To submit inquiries related to Otsuka clinical research, or to request access to individual participant data (IPD) associated with any Otsuka clinical trial, please visit https://clinical-trials.otsuka.com/. For all approved IPD access requests, Otsuka will share anonymized IPD on a remotely accessible data sharing platform.
