# Supplementary material for: Adjunctive brexpiprazole in patients with unresolved symptoms of depression on antidepressant treatment who are early in the disease course: post hoc analysis of randomized controlled trials
Source: Int J Neuropsychopharmacol. 2025 Jul 3;28(8):pyaf050. doi: 10.1093/ijnp/pyaf050 (PMC12982916; doi:10.1093/ijnp/pyaf050)
Supplement: Supplement_22-May-25_pyaf050 [file supplement_22-may-25_pyaf050.pdf]

Supplementary Material

Adjunctive Brexpiprazole in Patients With Unresolved Symptoms of Depression on Antidepressant Treatment Who are Early in the Disease Course: *Post Hoc* Analysis of Randomized Controlled Trials

Shivani Kapadia, PharmD,[a] Zhen Zhang, PhD,[a] Csilla Csoboth, MD, PhD,[b] Mehul Patel, PharmD,[a] Michael E. Thase, MD,[c] George I. Papakostas, MD[d]

[a]Otsuka Pharmaceutical Development & Commercialization Inc., Princeton, NJ, USA

[b]Lundbeck LLC, Deerfield, IL, USA

[c]Perelman School of Medicine, University of Pennsylvania and the Philadelphia Veterans Affairs Medical Center, Philadelphia, PA, USA

[d]Department of Psychiatry, Clinical Trials Network and Institute, Massachusetts General Hospital, Harvard Medical School, Boston, MA, USA

Contents

Supplementary Figure S1. Mean change from baseline to each week in MADRS total score, stratified by variables indicative of earlier and later disease course (2–3 mg analysis)

Supplementary Figure S2. Mean change from baseline to Week 6 in MADRS total score, stratified by variables indicative of earlier and later disease course (2 mg analysis)

Supplementary Table S1. Baseline demographic and clinical characteristics and assigned antidepressant treatments (2 mg analysis)

Supplementary Table S2. MADRS total score at baseline and treatment effect at Week 6, stratified by variables indicative of earlier and later disease course (2 mg analysis)

Supplementary Table S3. Summary of TEAEs, stratified by variables indicative of earlier and later disease course (2 mg analysis)

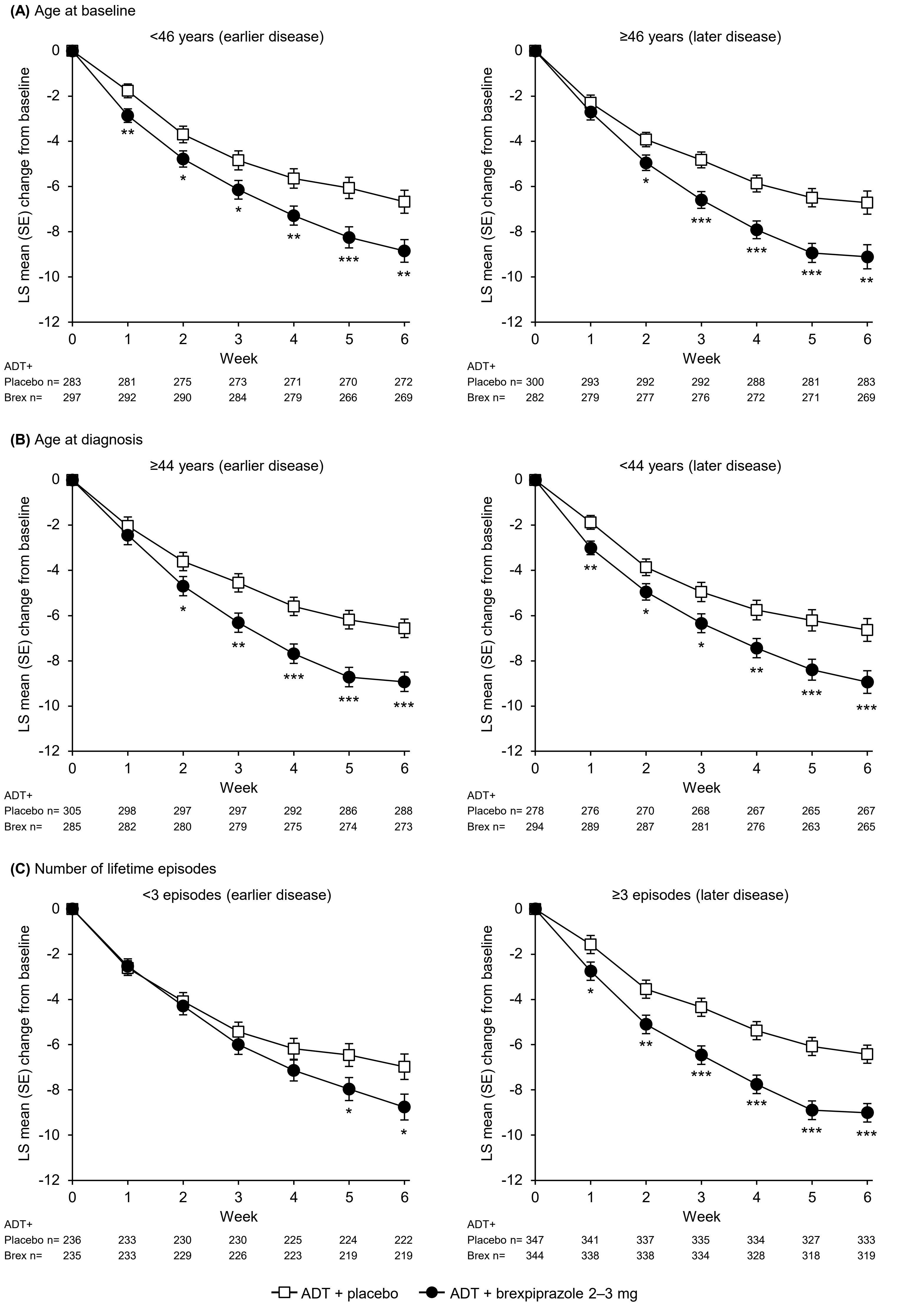
(A) Age at baseline
<46 years (earlier disease)
≥46 years (later disease)
LS mean (SE) change from baseline
Week
ADT+
Placebo n= 283 281 275 273 271 270 272
Brex n= 297 292 290 284 279 266 269
Placebo n= 300 293 292 292 288 281 283
Brex n= 282 279 277 276 272 271 269
(B) Age at diagnosis
≥44 years (earlier disease)
<44 years (later disease)
Placebo n= 305 298 297 297 292 286 288
Brex n= 285 282 280 279 275 274 273
Placebo n= 278 276 270 268 267 265 267
Brex n= 294 289 287 281 276 263 265
(C) Number of lifetime episodes
<3 episodes (earlier disease)
≥3 episodes (later disease)
Placebo n= 236 233 230 230 225 224 222
Brex n= 235 233 229 226 223 219 219
Placebo n= 347 341 337 335 334 327 333
Brex n= 344 338 338 334 328 318 319
ADT + placebo
ADT + brexpiprazole 2–3 mg

(D) Duration of current episode

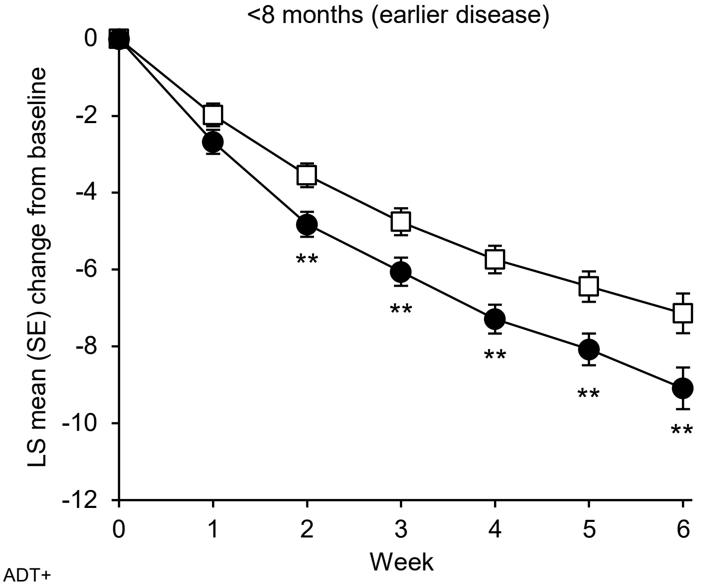

ADT+						
Placebo n= 272	268	267	264	259	261	260
Brex n= 256	252	250	248	248	241	242

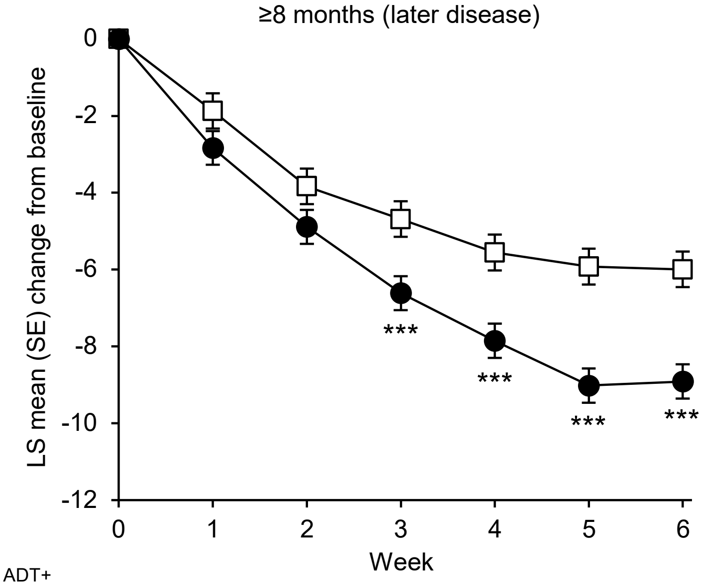

ADT+						
Placebo n= 311	306	300	301	300	290	295
Brex n= 323	319	317	312	303	296	296

(E) Number of prior ADTs

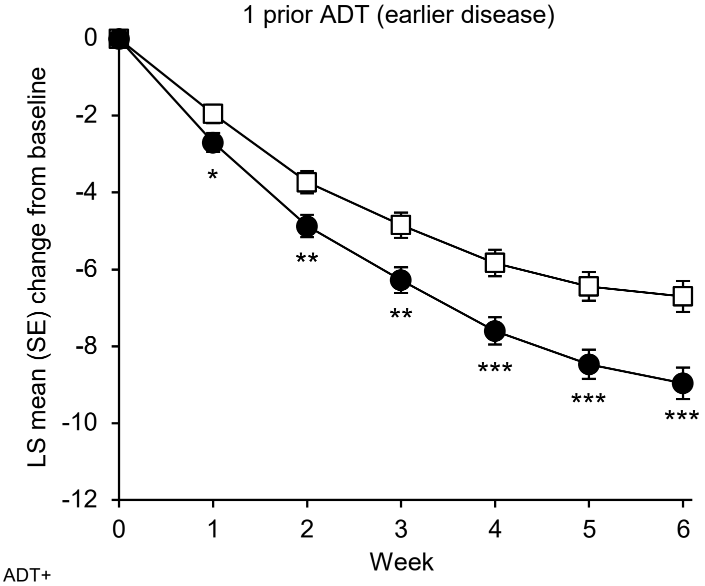

ADT+						
Placebo n= 469	463	458	456	450	441	445
Brex n= 467	460	457	451	445	431	433

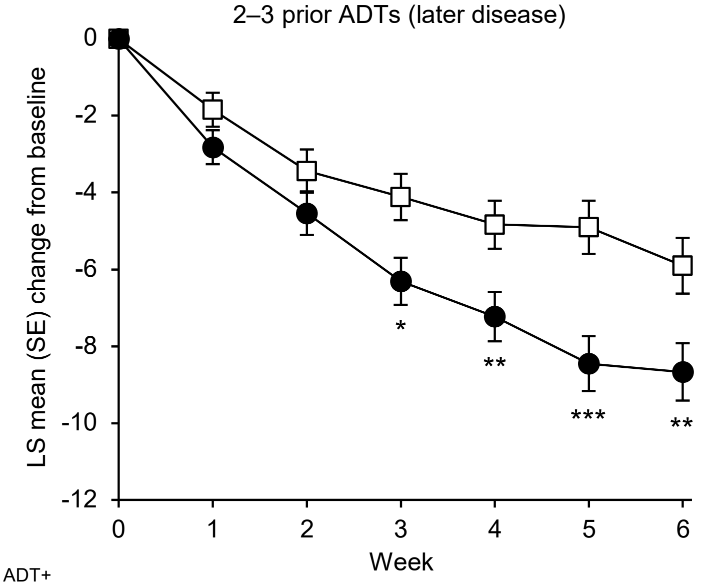

ADT+						
Placebo n= 114	111	109	109	109	110	110
Brex n= 106	105	104	103	100	100	99

ADT + placebo   ADT + brexpiprazole 2–3 mg

Supplementary Figure S1. Mean change from baseline each week in MADRS total score, stratified by variables indicative of earlier and later disease course (2–3 mg analysis). *p<0.05, **p<0.01, ***p<0.001 versus ADT + placebo. Abbreviations: ADT, antidepressant treatment; LS, least squares; MADRS, Montgomery–Åsberg Depression Rating Scale; SE, standard error.

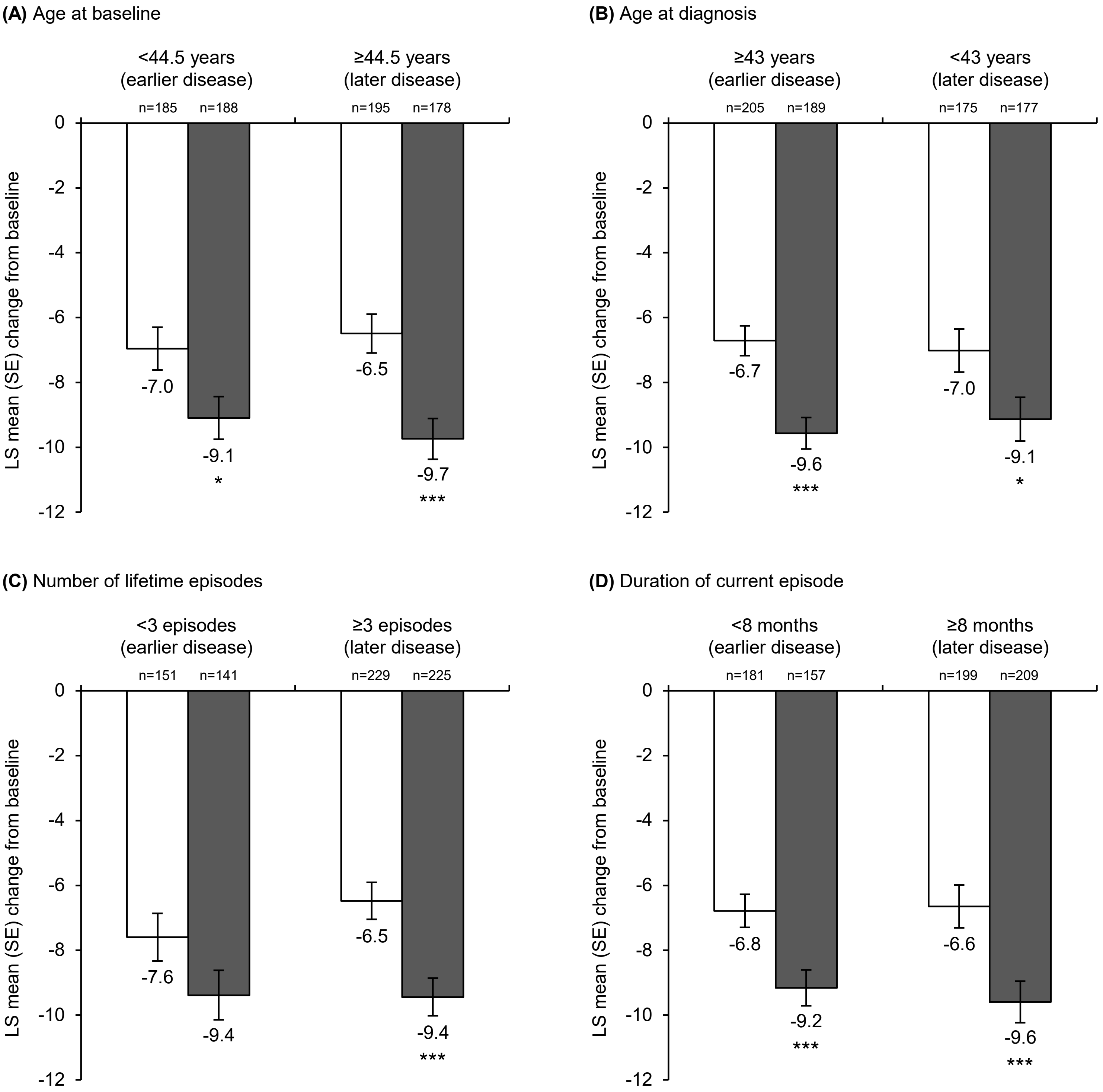

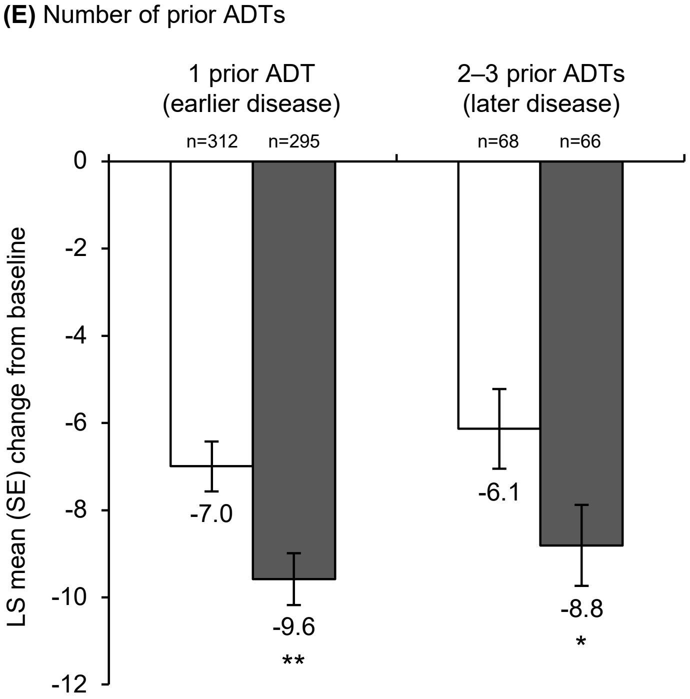

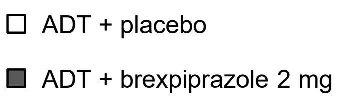

Supplementary Figure S2. Mean change from baseline to Week 6 in MADRS total score, stratified by variables indicative of earlier and later disease course (2 mg analysis). *p<0.05, **p<0.01, ***p<0.001 versus ADT + placebo. N-values are for baseline. Abbreviations: ADT, antidepressant treatment; LS, least squares; MADRS, Montgomery–Åsberg Depression Rating Scale; SE, standard error.

Supplementary Table S1. Baseline demographic and clinical characteristics and assigned antidepressant treatments (2 mg analysis)

Characteristic	ADT + placebo (n=380)	ADT + brexpiprazole 2 mg (n=366)
Age (years), mean (SD)	43.8 (12.1)	43.5 (12.0)
Sex, n (%)		
Female	271 (71.3)	266 (72.7)
Male	109 (28.7)	100 (27.3)
BMI (kg/m^2), mean (SD)	29.7 (7.1)	29.6 (6.8)
Race, n (%)		
White	326 (85.8)	314 (85.8)
Other[a]	54 (14.2)	52 (14.2)
Duration of current episode (months), mean (SD)	16.9 (36.2)	13.5 (14.4)
Number of lifetime episodes, mean (SD)	3.5 (2.7)	3.4 (2.6)
Number of prior ADTs at screening, n (%)[b]		
1	312 (82.1)	295 (81.7)
2	59 (15.5)	60 (16.6)
3	9 (2.4)	6 (1.7)
MADRS total score, mean (SD)	26.7 (6.0)	27.0 (5.7)
CGI-S score, mean (SD)	4.3 (0.6)	4.2 (0.6)
Assigned ADT, n (%)		
Escitalopram	74 (19.5)	75 (20.5)
Fluoxetine	58 (15.3)	59 (16.1)
Paroxetine CR	48 (12.6)	52 (14.2)
Sertraline	62 (16.3)	51 (13.9)
Duloxetine	80 (21.1)	62 (16.9)
Venlafaxine XR	58 (15.3)	67 (18.3)

[a]Including American Indian or Alaska Native, Asian, Black or African American, Native Hawaiian or Other Pacific Islander, and other non-specified (US Census Bureau classifications).

[b]Number of prior ADTs at screening was missing for 5 patients.

Abbreviations: ADT, antidepressant treatment; BMI, body mass index; CGI-S, Clinical Global Impressions – Severity of illness; CR, controlled release; MADRS, Montgomery–Åsberg Depression Rating Scale; SD, standard deviation; XR, extended-release.

Supplementary Table S2. MADRS total score at baseline and treatment effect at Week 6, stratified by variables indicative of earlier and later disease course (2 mg analysis)

Subgroup	N		Mean (SD) at baseline		Treatment difference at Week 6 (ADT + brex vs. ADT + placebo)	
	ADT + placebo	ADT + brex 2 mg	ADT + placebo	ADT + brex 2 mg	LS mean (95% CI)	p-value
Age at baseline						
<44.5 years (earlier disease)	185	188	26.7 (6.0)	26.9 (5.4)	-2.14 (-3.88 to -0.39)	0.016
≥44.5 years (later disease)	195	178	26.7 (5.9)	27.0 (6.0)	-3.25 (-4.89 to -1.60)	<0.001
Age at diagnosis						
≥43 years (earlier disease)	205	189	26.5 (6.0)	26.9 (5.8)	-2.85 (-4.07 to -1.63)	<0.001
<43 years (later disease)	175	177	27.0 (5.9)	27.0 (5.6)	-2.11 (-3.90 to -0.33)	0.021
Number of lifetime episodes						
<3 episodes (earlier disease)	151	141	26.7 (6.2)	26.5 (5.6)	-1.79 (-3.80 to 0.22)	0.081
≥3 episodes (later disease)	229	225	26.7 (5.8)	27.2 (5.8)	-2.97 (-4.48 to -1.45)	<0.001
Duration of current episode						
<8 months (earlier disease)	181	157	25.6 (5.8)	25.6 (5.7)	-2.37 (-3.71 to -1.04)	0.001
≥8 months (later disease)	199	209	27.7 (5.9)	28.0 (5.4)	-2.95 (-4.65 to -1.24)	0.001
Number of prior ADTs						
1 prior ADT (earlier disease)	312	295	26.7 (5.8)	27.2 (5.7)	-2.58 (-4.13 to -1.04)	0.001
2–3 prior ADTs (later disease)	68	66	27.0 (6.7)	25.9 (5.5)	-2.67 (-5.26 to -0.09)	0.043

Abbreviations: ADT, antidepressant treatment; brex, brexpiprazole; CI, confidence interval; LS, least squares; MADRS, Montgomery–Åsberg Depression Rating Scale; SD, standard deviation.

Supplementary Table S3. Summary of TEAEs, stratified by variables indicative of earlier and later disease course (2 mg analysis)

Subgroup	N		≥1 TEAE		≥1 EPS-related TEAE		Increased body weight TEAE	
	ADT + placebo	ADT + brex 2 mg	ADT + placebo	ADT + brex 2 mg	ADT + placebo	ADT + brex 2 mg	ADT + placebo	ADT + brex 2 mg
Age at baseline								
<44.5 years (earlier disease)	185	188	95 (51.4)	115 (61.2)	12 (6.5)	26 (13.8)	5 (2.7)	11 (5.9)
≥44.5 years (later disease)	195	178	89 (45.6)	103 (57.9)	7 (3.6)	22 (12.4)	2 (1.0)	13 (7.3)
Age at diagnosis								
≥43 years (earlier disease)	205	189	94 (45.9)	109 (57.7)	7 (3.4)	22 (11.6)	3 (1.5)	15 (7.9)
<43 years (later disease)	175	177	90 (51.4)	109 (61.6)	12 (6.9)	26 (14.7)	4 (2.3)	9 (5.1)
Number of lifetime episodes								
<3 episodes (earlier disease)	151	141	80 (53.0)	74 (52.5)	9 (6.0)	20 (14.2)	3 (2.0)	8 (5.7)
≥3 episodes (later disease)	229	225	104 (45.4)	144 (64.0)	10 (4.4)	28 (12.4)	4 (1.7)	16 (7.1)
Duration of current episode								
<8 months (earlier disease)	181	157	79 (43.6)	78 (49.7)	15 (8.3)	15 (9.6)	4 (2.2)	10 (6.4)
≥8 months (later disease)	199	209	105 (52.8)	140 (67.0)	4 (2.0)	33 (15.8)	3 (1.5)	14 (6.7)
Number of prior ADTs								
1 prior ADT (earlier disease)	312	295	150 (48.1)	171 (58.0)	16 (5.1)	38 (12.9)	6 (1.9)	17 (5.8)
2–3 prior ADTs (later disease)	68	66	34 (50.0)	42 (63.6)	3 (4.4)	9 (13.6)	1 (1.5)	5 (7.6)

Data are n (%).

Abbreviations: ADT, antidepressant treatment; brex, brexpiprazole; EPS, extrapyramidal symptom; TEAE, treatment-emergent adverse event.
